# EPIG-Seq: extracting patterns and identifying co-expressed genes from RNA-Seq data

**DOI:** 10.1186/s12864-016-2584-7

**Published:** 2016-03-22

**Authors:** Jianying Li, Pierre R. Bushel

**Affiliations:** Integrative Bioinformatics Group, National Institute of Environmental Health Sciences, Research Triangle Park, NC 27709 USA; Microarray and Genome Informatics Group, National Institute of Environmental Health Sciences, Research Triangle Park, NC 27709 USA; Biostatistics and Computational Biology Branch, National Institute of Environmental Health Sciences, 111 T.W. Alexander Drive, P.O. Box 12233, Research Triangle Park, NC 27709 USA; Kelly Government Solutions, Research Triangle Park, NC 27709 USA

**Keywords:** EPIG-Seq, Gene expression, RNA-Seq, Clustering, Pattern analysis, Toxicogenomics, Cancer

## Abstract

**Background:**

RNA sequencing (RNA-Seq) measures genome-wide gene expression. RNA-Seq data is count-based rendering normal distribution models for analysis inappropriate. Normalization of RNA-Seq data to transform the data has limitations which can adversely impact the analysis. Furthermore, there are a few count-based methods for analysis of RNA-Seq data but they are essentially for pairwise analysis of treatment groups or multiclasses but not pattern-based to identify co-expressed genes.

**Results:**

We adapted our extracting patterns and identifying genes methodology for RNA-Seq (EPIG-Seq) count data. The software uses count-based correlation to measure similarity between genes, quasi-Poisson modelling to estimate dispersion in the data and a location parameter to indicate magnitude of differential expression. EPIG-Seq is different than any other software currently available for pattern analysis of RNA-Seq data in that EPIG-Seq 1) uses count level data and supports cases of inflated zeros, 2) identifies statistically significant clusters of genes that are co-expressed across experimental conditions, 3) takes into account dispersion in the replicate data and 4) provides reliable results even with small sample sizes. EPIG-Seq operates in two steps: 1) extract the pattern profiles from data as seeds for clustering co-expressed genes and 2) cluster the genes to the pattern seeds and compute statistical significance of the pattern of co-expressed genes. EPIG-Seq provides a table of the genes with bootstrapped p-values and profile plots of the patterns of co-expressed genes. In addition, EPIG-Seq provides a heat map and principal component dimension reduction plot of the clustered genes as visual aids. We demonstrate the utility of EPIG-Seq through the analysis of toxicogenomics and cancer data sets to identify biologically relevant co-expressed genes. EPIG-Seq is available at: sourceforge.net/projects/epig-seq.

**Conclusions:**

EPIG-Seq is unlike any other software currently available for pattern analysis of RNA-Seq count level data across experimental groups. Using the EPIG-Seq software to analyze RNA-Seq count data across biological conditions permits the ability to extract biologically meaningful co-expressed genes associated with coordinated regulation.

**Electronic supplementary material:**

The online version of this article (doi:10.1186/s12864-016-2584-7) contains supplementary material, which is available to authorized users.

## Background

The advantages of RNA-sequencing (RNA-Seq) over microarray technology to measure gene expression have been reported recently [1–3]. Methods have been developed to analyze RNA-Seq data based on normalization of read counts or using raw count data [4–6]. Advantages of normalization are that it adjusts the data according to sequencing library size, accounts for the length of transcripts and allows for the use of analysis tools originally designed for microarray data. However, normalized RNA-Seq data or transformation of count data has limitations [7–10] which can adversely impact the analysis. Alternatively, using raw read counts circumvents the shortcomings of normalization but requires modelling of the data to estimate dispersion, accounting for library size and filtering to avoid cases of inflated zeros. In particular, statistical models of count data based on Poisson, beta- or negative-binomial distributions can be severely impacted by outliers in the data [11–13]. Unfortunately, there is a paucity of methodologies that can identify correlated gene expression patterns from RNA-Seq count data across biological conditions (i.e., time course, dose response, factorial study designs) [14]. Such paucity also limits the ability to cross-examine RNA-Seq and microarray analysis through comparable statistical measures, which can lead to discrepancies in data interpretation between these techniques.

We adapted the extracting patterns and identifying co-expressed genes (EPIG) methodology [15] for the identification of co-expressed genes from RNA-Seq data (EPIG-Seq). In the EPIG-Seq software patterns of gene expression across experimental groups are determined using a similarity measure for count data [16] to ascertain correlation between expression profiles, a quasi-Poisson model [13] to estimate dispersion in the data and a location parameter as a measure of the magnitude of difference between experimental conditions and control/baseline. EPIG-Seq then clusters each gene expression profile to the pattern for which it has the highest correlation. EPIG-Seq is different than any other software currently available for pattern analysis of RNA-Seq data in that EPIG-Seq identifies statistically significant clusters of co-expressed genes using count level data with or without inflated zeros. Furthermore, EPIG-Seq provides reliable results by taking into account dispersion in the data and defaulting to a robust/non-parametric magnitude of fold change estimator when sample sizes are small. We demonstrate the utility of EPIG-Seq by analyzing publicly available RNA-Seq data sets from the SEquence Quality Control (SEQC) toxicogenomics [1] arm of the MicroArray Quality Control (MAQC) consortium and from The Cancer Genome Atlas (TCGA) [17] breast cancer data portal. Using EPIG-Seq we identify several co-expressed genes related to modes of action (MOAs) of the chemical agents in the toxicogenomics data set and we also extract co-expressed genes that are being explored as molecular targets for breast cancer. Finally, EPIG-Seq has a user-friendly interface, it is also platform independent and provides a heat map, pattern profile plots and a principal component analysis dimension reduction plot of the clustered co-expressed genes as visual aids.

## Implementation

### Correlation

A compiled RNA-Seq gene expression data set consists of a 2-dimensional matrix where each row represents a gene expression profile and each column represents a sample. We denote x_*ij*_ as the count of reads from sample *j* mapped to gene *i* and *x*_*kj*_ as the count of reads from sample *j* mapped to gene *k*. To measure the count level correlation between two gene profiles, EPIG-Seq uses the *CY*_*s*_ similarity measure for count data previously defined as:

$$ C{Y}_{s_{ik}}=1-\frac{\mathrm{observed}\kern0.5em C{Y}_{d_{ik}}}{\mathrm{maximum}\kern0.5em C{Y}_{d_{ik}}} $$

where

$$ \mathrm{observed}\kern0.5em C{Y}_{d_{ik}}={\displaystyle \sum_{j=1}^a\left(\frac{\left({x}_{ij}+{x}_{kj}\right) \log \left(\frac{x_{ij}+{x}_{kj}}{2}\right)-{x}_{ij} \log {x}_{kj}-{x}_{kj} \log {x}_{ij}}{x_{ij}+{x}_{kj}}\right)}, $$

*a* is the total number of samples with read counts mapped to either profile and log is the natural logarithm. As such, $C{Y}_{s_{ik}}=0$ when two profiles are totally different and $C{Y}_{s_{ik}}=1$ when the two are identical. The *CY*_*s*_ similarity measure is similar to other distance or similarity metric (i.e., Horn’s index [18]) and was originally used for assessing variation in species abundance in ecological and environmental monitoring. Its nomenclature originates from initials of the lead author introducing its use and is shown to outperform other similarity measures on species abundance count data [16, 19]. CYs works better than the Spearman rank correlation coefficient when the expression of the genes within all the groups is relatively the same except in the control/baseline/reference. The Spearman rank correlation coefficient treats these as ties and hence does not allow responsive but invariant patterns across treatment groups to be extracted. Further details of the computation of the *CY*_*s*_ correlation including the maximization of *CY*_*d*_ (the dissimilarity measure) are available in the Additional file 1.

### Magnitude of change

In EPIG-Seq, the strength of a gene expression profile’s signal is defined according to the value of the test-statistic location parameter obtained from a Wilcoxon rank sum non-parametric test [20] measuring the difference between the ranks of the expression of the genes in sample *X* vs those in sample *Y*. Here, sample *X* is the biological replicates from the treated, perturbed or diseased group and sample *Y* is the biological replicates from the controls. When the sample size for each group is small, the approximated Z-statistic from the Wilcoxon rank sum test can be spurious. In such a case, EPIG-Seq defaults to measure the strength of the *g*th gene’s differential expression according to the value of the Hodges-Lehmann location parameter estimator ${\widehat{\varDelta}}_g$ for the difference between two groups of independent samples [20]. See the Additional file 1 for further details of the magnitude of change.

### Dispersion

For each *g*th gene expression profile, EPIG-Seq estimates the dispersion parameter *θ* using a quasi-Poisson regression to model the data. The quasi-Poisson likelihood model is commonly used for overdispersed count data as it incorporates *θ* into the Poisson model such that that V(*Y*_*g*_) = *μ*_*g*_*θ*_*g*_ [21]. Further details of the dispersion and count data modelling are available in the Additional file 1.

### Clustering of gene expression profiles to patterns

EPIG-Seq runs in two steps. The first step (pattern profile determination) involves pairwise correlations of all the genes and tallying those which have a CY_s_ correlation > = Rt1 and at least Mt-1 highly correlated genes. The genes that meet these criteria are further filtered according to those with a magnitude of change ≤ St1 and dispersion < or > 5 % in each tail of the distribution. The remaining genes are defined as the pattern profiles for co-expression clustering. In step two (clustering of the genes to the pattern profiles), genes are clustered to the patterns using initialization and recursion strategies that are typical in standard clustering methodologies [22, 23]. The $C{Y}_{s_{ik}}$ measure is used to correlate the *i*th gene to the *k*th pattern profile. The gene is assigned to the pattern to which it has the highest similarity to (i.e. a CY_s_ correlation > = Rt2). Once all the genes are assigned, a representative gene for each pattern is determined by a pattern correlation score (PCS) and the clustering continues recursively until there are no more movement of genes or the # of moves = 100. See the Additional file 1 for further details of the PCS, the clustering of gene expression profiles to patterns and the EPIG-Seq algorithm pseudo code.

### Assessing the significance of the clustering

To assess the significance of the clustering of the co-expressed genes to the extracted patterns, we performed *B* number of bootstrapped assignments of *P* random gene profiles to a pattern and compute the PCS each time to compare to the observed PCS for that pattern. Briefly, for *B* times and for a given pattern containing *P* gene profiles, we randomly select *P* number of gene profiles from the data set. Then, for the selected *P* random profiles, we compute the bootstrapped PCS. The *p*-value for a pattern is computed as the number (*n*) of times one of these bootstrapped scores is greater than the observed score. Thus, *p*-value = *n*/*B*. Statistical significance of each co-expressed gene is determined by the *p*-value from the generalized linear model of the count data. The resulting patterns represent statistically significant clusters of genes that are biologically meaningful due to their shared co-expression across the treatment groups/biological conditions. In other words, the genes respond similarly. See the Additional file 1 for further details of the count data modelling of RNA-Seq data.

### Publicly available data

#### TCGA breast cancer RNA-Seq data

The Cancer Genome Atlas (TCGA) provides open access to genomic data acquired from various forms of cancer. Institutional review boards at each tissue source site reviewed protocols and consent documentation and approved submission of cases to TCGA. Cases were staged according to the American Joint Committee on Cancer (AJCC) staging system. To evaluate EPIG-Seq’s ability to extract biologically relevant patterns of gene expression, we downloaded count-level breast ductal carcinoma RNA-Seq data [17] produced on the Illumina GAII sequencer and processed as described in the Additional file 1. The RNA-Seq data was obtained from the specimens of patients with appropriate informed consent pre-existing with the TCGA repository. The breast tumor samples were classified by the mRNA subtypes [24–26]. We only used data from the following four subtypes: luminal A, luminal B, Her2-enriched and basal-like. The latter subtype is often consider aggressive and to have a poorer prognosis. Patterns extracted with co-expressed genes exhibiting varied expression within the basal-like subtype apart from the other subtypes would be of interest for molecular profiling of the tumor. To generate 4 separate “sampled” data sets of reasonable size (*n* = 50), for each one, we randomly selected 10 lanes from each tumor subtype plus 10 lanes from normal breast tissue.

#### Toxicogenomics RNA-Seq data

The cascade of biochemical and molecular initiating events (MIEs; i.e., the biological targets of a chemical) following a toxicological exposure is referred to as the MOA. RNA-Seq data from the MAQC phase III SEQC crowd source toxicogenomics (TGxSEQC) effort [1] was acquired from the livers of male Sprague-Dawley rats exposed to chemicals that share a MOA and is available in the National Center for Biotechnology Information Sequence Read Archive (SRA) [27] under accession number SRP039021 and the Gene Expression Omnibus under accession number GSE55347 . We used the training data set containing RNA-Seq data from 15 chemicals or vehicle and route matched controls. Animals were handled in accordance with United States Department of Agriculture and Code of Federal Regulations Animal Welfare Act (9 CFR Parts 1, 2, and 3). Sets of three chemicals share one of five MOAs. Three MOAs are associated with well-defined receptor-mediated processes—peroxisome proliferator-activated receptor alpha (PPARA), orphan nuclear hormone receptors (CAR/PXR) and aryl hydrocarbon receptor (AhR). The other two are non-receptor-mediated—DNA damage (DNA_Damage) or cytotoxicity (Cytotoxic). Patterns extracted with genes exhibiting varied co-expression in one or more MOAs may elucidate MIEs shared between chemicals. Specific details of the study design and sample collection are available in the TGxSEQC publication. Further details of the alignment of the RNA-Seq reads and bioinformatics pipeline are available in the Additional file 1.

### Software usability

Using EPIG-Seq to identify patterns of gene expression and to identify co-expressed gene is straight forward and simple using the graphical user interface (GUI). The data format for analysis requires a tab-delimited text file with the 1st row containing the labels of the groups (*n* > 3) that the samples belong to (one group must be of samples that are controls/baseline/background) and the 2nd row containing the total mapped reads for each sample. The latter is used for visualization of the results as log base 2 ratio reads per million (RPM) data. The 1^st^ column must contain unique gene IDs and the data in the remaining cells the read counts (as integers) per gene.

EPIG-Seq analysis proceeds in two steps (pattern identification and gene clustering), both of which have parameter setting for correlation of the genes and magnitude of differential gene expression change (in at least one group compared to the baseline/controls/background samples). Correlation is based on the CY_s_ measure with a higher value denoting more correlation. Magnitude of differential change is according to the Z-statistics from the Wilcoxon rank sum non-parametric tests of each comparator group to the baseline/controls/background group. Thus, the magnitude of change resembles the deviation from a standard normal distribution. For instance, a Z-statistic = 2 translates to an approximate probability of 0.05 that the gene expression is statistically different in the comparator group than the baseline/controls/background group. Since the CY_s_ measure doesn’t account for direct or anticorrelation, in EPIG-Seq the signs of the Z-statistics are used to constrain the directionality of the correlation.

Step 1 (pattern identification) has three additional user-defined parameters: 1) minimum number of gene profiles to make a pattern, 2) the correlation setting to weed out redundant patterns and 3) the number of central processing units (CPUs) to use for processing. Increasing the first two parameters will reduce the number of patterns extracted. Increasing the number of CPUs will substantially decrease the processing time as parallel computing is utilized. Lastly, step 1 has a gene dispersion threshold setting to discard gene profiles from pattern consideration if their dispersions are < or > 5 % in each tail of the distribution.

Step 2 (gene clustering) has an additional user-defined parameter for the number of bootstraps needed to compute the non-parametric *p*-value in determining significance of the patterns. Increasing the number of bootstraps will increase the reliability of the estimated *p*-value with a cost of a longer processing time. Finally, step 2 has a clustering iteration threshold equal to either a < 0.0001 difference between two successive recursive clusterings of the genes to the patterns or the clustering recursion proceeded for 100 iterations.

The main steps in the EPIG-Seq analysis proceeds as follows:

- Compute Z-statistics for each gene profile
- Perform pairwise correlations between gene profiles
- Extract patterns based on Step 1 parameters
- Remove redundant patterns
- Use the gene profiles with the top PCS from each unique pattern as the seeds for the gene profile clustering
- Compute the *p*-value for each gene profile
- Terminate clustering of profiles to patterns
- Compute *p*-values for the patterns
- Present the results in output files, figures and a table of co-expressed genes

## Results

### Development of EPIG-Seq

We patterned the development of EPIG-Seq to resemble the steps and components that comprise EPIG [15] for analyzing gene expression patterns from microarray data. As shown in Table 1, EPIG-Seq uses a *CY*_*s*_ measure, the magnitude of a Wilcoxon rank sum statistic and variance to mean ratio (VMR) for RNA-Seq count data. These provide several advantages of EPIG-Seq on the analysis of RNA-Seq data. First, it supports cases where the read count is zero. Second, since correlation across samples is used, EPIG-Seq is not affected by differences in total read count per sample/lane of RNA-Seq. Third, it supports the discrete Poisson distribution typical of RNA-Seq count data and uses a quasi-Poisson model to account for dispersion in the data. Finally, when within group sample sizes are small, it uses the robust and non-parametric Hodges-Lehmann estimator as the location parameter for the magnitude of gene expression change.

**Table 1:** EPIG-Seq configuration

Data type	Count level
Distribution assumption	Poisson
Correlation measurement	CYs
Spread of the data	Dispersion
Magnitude of change	Wilcoxon test Z-Statistic
Dynamic range	Variance-to-mean ratio (VMR)

**Fig. 1:**
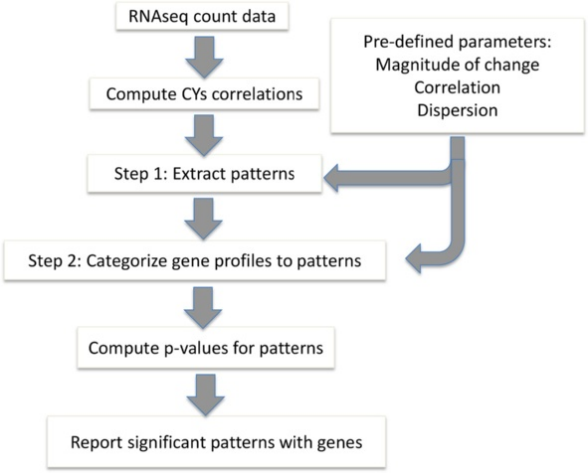
EPIG-Seq workflow. The workflow depicts the main steps of EPIG-Seq. The parameters are used in steps 1 and 2 to extract the patterns and cluster the genes respectively. The output is the statistically significant patterns with co-expressed genes

As shown in Fig. 1, the 1^st^ step in EPIG-Seq is to find all patterns in the data. Once the patterns are identified, in step 2, the gene expression profiles are clustered to the patterns to group co-expressed genes. Clustering is performed iteratively until the patterns with the co-expressed genes stabilize. The gene is assigned to the pattern to which it has the highest similarity to. Once all the genes are assigned, a representative gene for each of the patterns is determined by the PCS which is the highest median correlation to the other genes in the pattern.

**Fig. 2:**
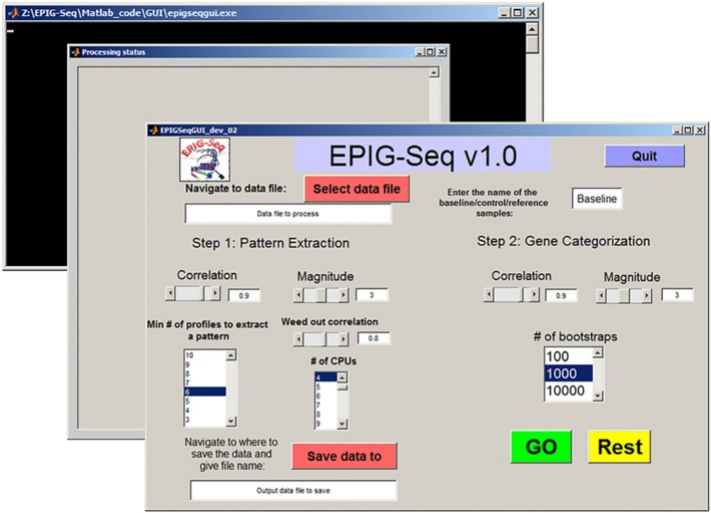
EPIG-Seq GUI. The EPIG-Seq GUI contains a main panel which allows users to define parameters for steps 1 and 2 of the analysis process. A dialog box displays the processing status and a command window displays the dependent processes running in the background

Figure 2 shows the GUI for EPIG-Seq. Default values for the parameters are preassigned for pattern extraction (step 1) and gene clustering (step 2) but can be changed to suit the analysis stringency (see Additional file 2: Figure S1A and B for optimization of parameters for EPIG-Seq steps 1 and 2 respectively using simulated data). The five patterns extracted from the simulated data illustrate the utility of EPIG-Seq to extract only the real responsive patterns (not the noisy one), identify co-expressed genes and group the samples (Additional file 3: Figure S2A, B and C respectively). Increasing the correlation will result in fewer patterns extracted and fewer genes clustered. Increasing the magnitude will require larger fold change responses cross the treatment groups. Increasing the weed out correlation will result in fewer redundant patterns extracted. The status of the EPIG-Seq processing of the data is monitored through a dialog box.

### EPIG-Seq co-expression analysis

**Fig. 3:**
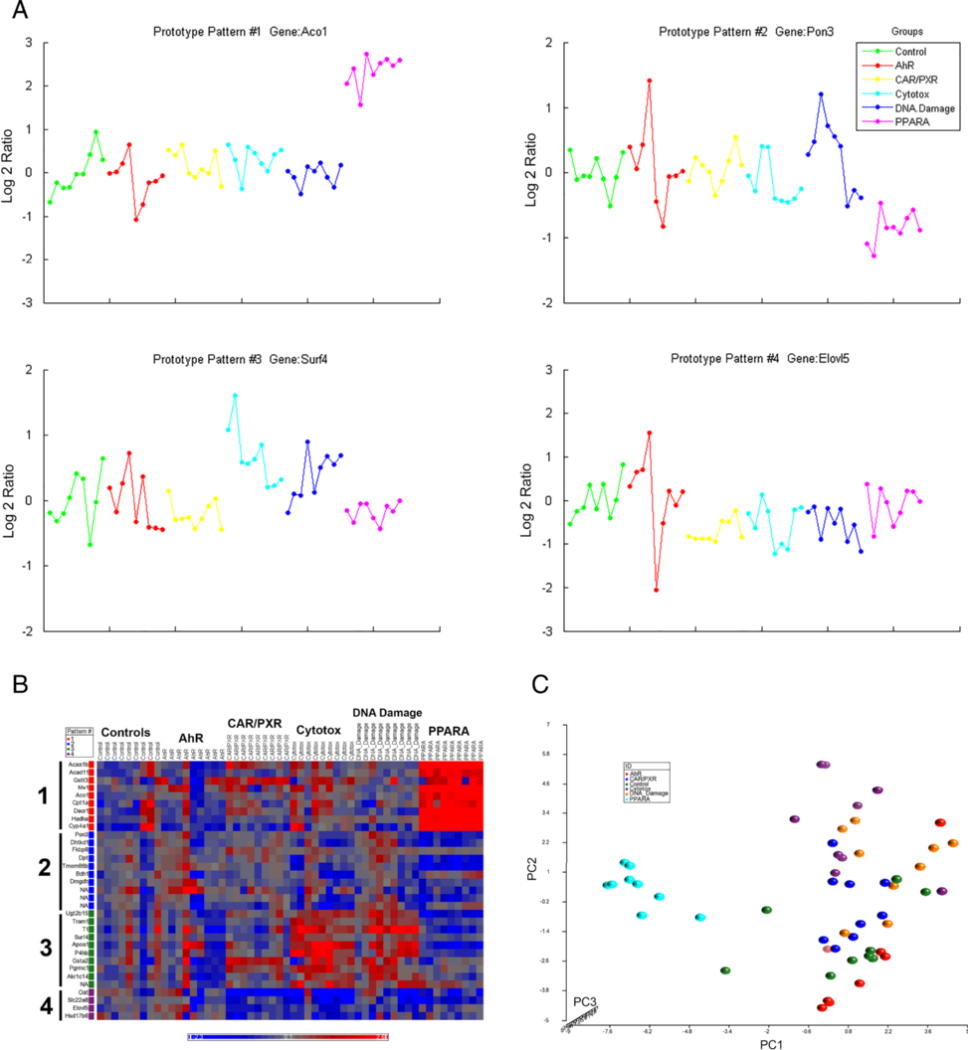
EPIG-Seq analysis of the toxicogenomics MOA data. **a** Thumbnail plots of the gene expression profiles that are the representatives (those with the highest PCS) of each of the extracted patterns from the toxicogenomics MOA data. The title of each thumbnail plot indicates the number of the pattern extracted and the gene symbol. MOA groups are color-coded as follows: Control (*green*), AhR 2 (*red*), CAR/PXR (*yellow*), Cytotox (*light blue*), DNA Damage (*blue*) and PPARA (*pink*), with 9 samples (groups of 3 biological replicates per chemical) in each. The y-axis is the log base 2 ratio of each sample data RPM relative to the average of the control. **b** The heat map representation of the genes clustered to the four extracted patterns from the EPIG-Seq analysis of the toxicogenomics MOA data. The symbols of the genes are shown to the left of the heat map with the 4 colors indicative of the pattern number assigned to. The columns indicate the chemicals within each of the MOA groups. The color scale represents the log base 2 ratio of each sample data relative to the average of the control. **c** PCA of the toxicogenomics MOA data using the CY_s_ correlation measures of the genes clustered to the patterns by EPIG-Seq. The groups are color-coded as denoted in the legend. The x-axis is PC1, the y-axis is PC2 and the z-axis is PC3

To demonstrate the utility of EPIG-Seq, we analyzed real RNA-Seq data. EPIG-Seq analysis of the MAQC Toxicogenomics data set (samples of RNA from the livers of rats exposed to chemicals that share a common mode of action) extracted four patterns of co-expressed genes when using *CY*_*s*_ and St for steps 1 and 2 equal to 0.8 and 1 respectively and percentile (PCT) = 5 % as parameter settings (Fig. 3a). The pattern representatives (genes used as seeds) are shown (Aco1, Pon3, Surf4 and Elovl5). The more impacting treatments in terms of gene regulation were seen from the exposure to PPARA chemicals (Fig. 3b). This biased co-expression of genes is expected as the chemicals with the PPARA MOA (Bezafibrate, Nafenopin and Pirinixic acid) were shown to have about 59 % more differential expression (~6,500 on average) than the chemicals in the other MOAs (~4,100 on average) [1]. In other words, the PPARA MOA chemicals elicit a stronger transcriptional response than the other MOA chemicals. Also, despite the heterogeneity in the data and the three chemicals per MOA, EPIG-Seq was still able to extract discernable patterns. In pattern 1, the genes were upregulated by PPARA chemical treatments and unchanged by the other treatments. Pattern 2 depicts the converse. Pattern 3 shows a down-regulation by PPARA chemicals but a slight up-regulation of genes by CAR/PXR, cytotoxic agents and DNA damage toxicants. Pattern 4 illustrates the down-regulation of all the MOA chemicals except for AhR. There were 33 genes clustered in total (Table 2). Gene ontology biological processes enrichment of the genes reveals regulation of fatty acid metabolism, oxidation/reduction and homeostatic processes impacted by co-expressed genes in patterns 1, 2 and 3 respectively (Table 3). PCA of the 33 genes confirms that the treatments by the PPARA chemicals support the notion that the treated samples from the treatment of chemicals in this MOA are very different from the others (Fig. 3c).

**Table 2:** Co-expressed genes from EPIG-Seq analysis of the MOA RNA-Seq data

Pattern #	Genebank Acc. #	Symbol	Description
1	NM_001040019	Acaa1b	Acetyl-Coenzyme A acyltransferase 1B
1	NM_001108181	Acad11	Acyl-CoA dehydrogenase family, member 11
1	NM_001137643	Gstt3	Glutathione S-transferase, theta 3
1	NM_012600	Me1	Malic enzyme 1, NADP(+)-dependent, cytosolic
1	NM_017321	Aco1	Aconitase 1, soluble
1	NM_031559	Cpt1a	Carnitine palmitoyltransferase 1a, liver
1	NM_057197	Decr1	2,4-dienoyl CoA reductase 1, mitochondrial
1	NM_130826	Hadha	Hydroxyacyl-CoA dehydrogenase/3-ketoacyl-CoA thiolase/enoyl-CoA hydratase (trifunctional protein), alpha subunit
1	NM_175837	Cyp4a1	Cytochrome P450, family 4, subfamily a, polypeptide 1
2	NM_001004086	Pon3	Paraoxonase 3
2	NM_001025720	Dhtkd1	Dehydrogenase E1 and transketolase domain containing 1
2	NM_001037180	Fkbp8	FK506 binding protein 8
2	NM_001105965	Dpt	Dermatopontin
2	NM_001109604	Tmem86b	Transmembrane protein 86B
2	NM_053995	Bdh1	3-hydroxybutyrate dehydrogenase, type 1
2	NM_139102	Dmgdh	Dimethylglycine dehydrogenase
2	XM_002728268	NA	NA
2	XM_002728512	NA	NA
2	XM_002728876	NA	NA
3	NM_001004271	Ugt2b15	UDP glucuronosyltransferase 2 family, polypeptide B15
3	NM_001007701	Tram1	Translocation associated membrane protein 1
3	NM_001013110	Tf	Transferrin
3	NM_001033868	Surf4	Surfeit 4
3	NM_012738	Apoa1	Apolipoprotein A-I
3	NM_012998	P4hb	Prolyl 4-hydroxylase, beta polypeptide
3	NM_017013	Gsta2	Glutathione S-transferase alpha 2
3	NM_021766	Pgrmc1	Progesterone receptor membrane component 1
3	NM_138547	Akr1c14	Aldo-keto reductase family 1, member C14
3	NM_175843	NA	NA
4	NM_022521	Oat	Ornithine aminotransferase
4	NM_031332	Slc22a8	Solute carrier family 22 (organic anion transporter), member 8
4	NM_134382	Elovl5	ELOVL fatty acid elongase 5
4	NM_173305	Hsd17b6	Hydroxysteroid (17-beta) dehydrogenase 6

**Table 3:** GO biological processes of MOA clustered genes

Pattern #	# of Genes	Top GOBP	*p*-value	FDR
1	9	GO:0006631 - Fatty acid metabolic process	3.8E-06	4.4E-03
2	10	GO:0055114 - Oxidation reduction process	2.3E-02	2.1E + 01
3	10	GO:0042592 - Homeostatic process	6.0E-02	5.5E + 01
4	4	-	-	-

*GOBP* Gene Ontology biological processes filtered to remove very broad GO terms

As another example of EPIG-Seq’s utility, we analyzed TCGA breast cancer RNA-seq data derived from 10 randomly selected lanes from each “subtype” to construct balanced data sets of the four breast cancer subtypes plus normal breast tissue as a control. Using *CY*_*s*_ and St for steps 1 and 2 equal to 0.8 and 2 respectively and PCT = 5 % as parameter settings, EPIG-seq extracted four or six patterns from either of the TCGA sampled data with between 192 and 344 genes in total per sampled set (Table 4 and Additional file 4: Tables S1-S4). The general silhouette of the genes reveals that the patterns are relatively cohesive and separated well. Since the data sets were randomly selected from the pool, there is stochastic variation in the data that introduces variability in the results. From the clustering comparison (Table 5), good adjusted mutual information (AMI) agreement was observed, although comparisons between data set 1 and data sets 3 and 4 yielded low scores of 0.524 and 0.452 respectively. This points to the possibility that sampled data set 1 is somewhat of an outlier.

**Table 4:** EPIG-Seq clustering cohesiveness of patterns extracted from the TCGA breast cancer sampled data

Sample #	GS	MS	# of Patterns	# of Genes
1	0.31	0.54	6	192
2	0.37	0.51	4	169
3	0.21	0.52	6	344
4	0.41	0.59	4	197

*GS* general silhouette, *MS* Maximal silhouette

**Table 5:** Agreement of clusters extracted from the TCGA breast cancer sampled data

Samples compared	Agreement
1 vs 2	0.770
1 vs 3	0.524
1 vs 4	0.452
2 vs 3	0.691
2 vs 4	0.751
3 vs 4	0.500

All comparisons based on AMI except for those with sample 2 where concordance was used

## Discussion

RNA-Seq has its advantages over microarray gene expression analysis. Tools for analysis of RNA-Seq data primarily test pair-wise comparisons or are analysis of variance (ANOVA)-like but they compute on the count data. We developed a version of our EPIG tool for microarray gene expression to support RNA-Seq count data (EPIG-Seq). An advantage of EPIG-Seq is that gene expression profiles from the RNA-Seq data are analyzed across a set of treatment conditions or series of perturbations. In addition, EPIG-Seq supports data with inflated zeros and that is overdispersed. Using count-based correlation to measure similarity between gene expression profiles, quasi-Poisson modelling to estimate dispersion in the data and a location parameter to indicate the strength of differential expression, EPIG-Seq clustered genes to the statistically significant patterns that they correlate with across conditions. Other tools for analysis of RNA-Seq count data are not directly comparable to EPIG-Seq since they don’t correlate gene expression across the treatment [4, 11, 12, 28].

Analysis of EPIG-Seq on real data yielded biologically meaningful results about the co-regulation of genes. For example, analysis of the MAQC Toxicogenomics data set with RNA samples of livers from rats exposed to chemicals that share a MOA, EPIG-Seq identified genes in patterns that are key toxicological processes in metabolism and oxidation/reduction (Table 3). For instance, in pattern 1 where the genes are up-regulated by the PPARA MOA chemicals and essentially unchanged in the other treatments (Fig. 3a and b), Cytochrome P450, Family 4, Subfamily A, Polypeptide 1 (Cyp4a1 -PPARA inducible) is one of the hallmark PPARA responsive genes co-expressed [29]. In the rat liver, Cyp4a1 is induced by binding of peroxisome proliferator ligands to the PPARA receptor [30]. Furthermore, motif analysis [31] of the −1000 to +1000 DNA sequence region of the nine genes in pattern 4 uncovered an enriched transcription factor binding site (TATAACA) as overrepresented with a *p*-value = 4.17×10^−4^.

**Fig. 4:**
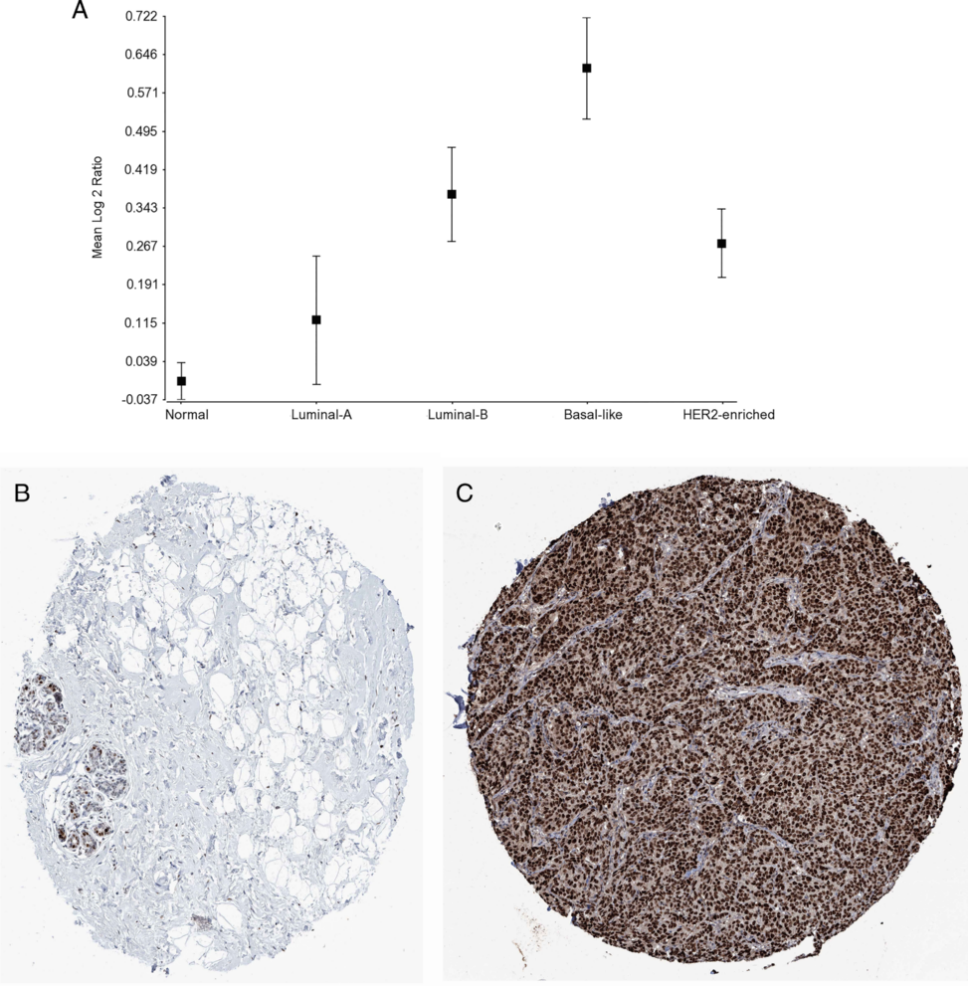
PCNA expression. **a** Gene expression of PCNA in TCGA normal and breast cancer samples. The x-axis denotes the breast cancer tumor subtype. The y-axis is the average of the log base 2 ratio of PCNA in each tumor subtype relative to the average of the normal samples. Standard error bars are shown for each data point. **b** PCNA protein immunohistochemistry staining of normal breast tissue with benign adenomas from a female age 23 (ID: 2773) and using the HPA030522 antibody. **c** PCNA protein immunohistochemistry staining of breast cancer tissue (ductal carcinoma) from a female age 55 (ID: 2773) and using the HPA030522 antibody

Analysis of the TCGA breast cancer data sample #3 produced DNA replication, intracellular protein transport, PPAR signaling, response to organic substances and translation elongation biological pathways as overrepresented categories (Table 6) commonly associated with breast cancer progression and metastasis [32, 33]. In particular, pattern #1 contains the up-regulation of proliferating cell nuclear antigen (PCNA) (Fig. 4a), topoisomerase (DNA) II (TOP2A) and S100 calcium binding protein A14 (S100A14) genes. In fact, PCNA, TOP2A and other genes in the patterns have been targets for breast cancer therapy [34, 35]. Interestingly, the Human Protein Atlas [36] contains immunohistochemistry staining of the PCNA protein (antibody HPA030522) in normal breast tissue (Fig. 4b) versus the overexpression in breast cancer ductal carcinoma (Fig. 4c) implicating the abundance of the protein in breast cancer tissue as a potential biomarker [37].

**Table 6:** Pathway enrichment of breast cancer co-expressed genes

Pattern #	# of Genes	Enriched Pathways (example of co-expressed genes)	*p*-value	FDR
1	45	GO:0006260 - DNA replication (PCNA, TOP2A, S100A14)	1.10E-05	1.70E-02
2	182	GO:0006886 - Intracellular protein transport (ERBB3, PTMS, SLC25A5, SLC9A3R1, SOX4, STAT1)	1.1E-06	1.8E-03
3	9	BST2, C17orf37, CEACAM6, IFI27, IFI6, MX1, OAS3, RAB31, TPX2	-	-
4	40	KEGG:03320 - Peroxisome proliferator-activated receptor signaling pathway (TRIM29, PDK4, FOSB, CD36)	1.0E-04	9.1E-02
5	41	GO:0010033 - Response to organic substance (ANXA1, CD34, EGR1, FOS, TGFBR2)	1.3E-04	2.0E-01
6	27	GO:0006414 - Translation elongation (CD59, ITGB1, ribosomal protein genes)	2.80E-05	3.90E-02

## Conclusions

EPIG-Seq is unlike any other software currently available for pattern analysis of RNA-Seq count level data across experimental groups. EPIG-Seq analysis of RNA-Seq count data across biological conditions permits the ability to extract biologically meaningful co-expressed genes associated with coordinated regulation. The approach leverages a count based correlation to identify patterns of expression across samples, accounts for the dispersion in the data and uses a location parameter to indicate magnitude of differential expression whether the sample size is large or small. EPIG-Seq analysis of TCGA human breast cancer RNA-Seq data extracts genes regulated across the various subtypes including PCNA, one of the key marker genes. EPIG-Seq analysis of a rat liver toxicogenomics RNA-Seq data set reveals genes that co-expressed across MOAs containing chemicals with similar MIEs such as PPAR antagonists and the Cyp4a1 PPAR-α inducible gene. Thus, using the EPIG-Seq software to analyze RNA-Seq count data across biological conditions permits the ability to extract biologically meaningful co-expressed genes associated with coordinated regulation.

## Availability and requirements

Project name: EPIG-Seq

Project home page: e.g. http://sourceforge.net/projects/epig-seq

Operating system(s): Windows and Linux

CPU architecture: Multicores recommended

Programming language: e.g. C and Java currently, R in future implementations

Other requirements: R version ≥ 3.1.2 and CRAN R package stats version 3.1.2 to fit a generalized linear model (glm)

License: GNU GPL-2 | GPL-3

Any restrictions to use by non-academics: License needed to distribute the programs containing code from R and for the Matlab MCRInstaller from MathWorks.

## Additional files

Additional file 1: Supplemental methods are more detailed descriptions of some of the computational methods used in EPIG-Seq. (DOCX 97 kb) Additional file 2: Figure S1. A and B are tiff image files of the plots illustrating the optimization of parameters for EPIG-Seq steps 1 and 2 respectively using simulated data. (TIF 19517 kb) Additional file 3: Figure S2. EPIG-Seq analysis of the simulated data. A) Thumbnail plots of the simulated gene profiles that are the representatives (those with the highest pattern correlation score) of each of the extracted patterns. B) Heat map representation of the patterns extracted and the simulated gene profiles clustered to each. C) PCA of the simulated gene profiles clustered to the patterns by EPIG-Seq using the CYs correlation measures. (JPG 3477 kb) Additional file 4: Tables S1-S4. Are Excel spreadsheets containing the EPIG-Seq clustered genes from the TCGA data samples 1 – 4 respectively. (XLSX 665 kb)

## Abbreviations

AhR: aryl hydrocarbon receptor
AMI: adjusted mutual information
ANOVA: analysis of variance
ARI: adjusted Rand Index
CAR/PXR: Constitutive Androstane Receptor/Pregnane X Receptor
CPUs: central processing units
Cyp4a1: Cytochrome P450, Family 4, Subfamily A, Polypeptide 1
CY_s_: correlation for count data
Cytotoxic: cytotoxicity
DNA_Damage: DNA damage
EPIG: extracting patterns and identifying co-expressed genes
EPIG-Seq: extracting patterns and identifying co-expressed genes from RNA-Seq data
GUI: graphical user interface
MAQC: MicroArray Quality Control
MI: mutual information
MIE: molecular initiating event
MOA: mode of action
Mt: number of profiles to form candidate patterns in EPIG-Seq step 1
NB: negative binomial
NCI: National Cancer Institute
PCA: principal component analysis
PCNA: proliferating cell nuclear antigen
PCS: pattern correlation score
PCT: percentile
PPARA: peroxisome proliferator-activated receptor alpha
RNA-Seq: RNA sequencing
RPM: reads per million
Rt1: CY_s_ correlation for EPIG-Seq step 1
Rt2: CY_s_ correlation for EPIG-Seq step 2
SEQC: SEquence Quality Control
SNR: signal to noise ratio
SRA: sequence read archive
St1: magnitude of fold change for EPIG-Seq step 1
St2: magnitude of fold change for EPIG-Seq step 2
TCGA: The Cancer Genome Atlas
TGxSEQC: Toxicogenomics SEQC
TOP2A: Topoisomerase (DNA) II
VMR: variance to mean ratio
