# Supplementary material for: EPIG-Seq: extracting patterns and identifying co-expressed genes from RNA-Seq data
Source: BMC Genomics. 2016 Mar 22;17:255. doi: 10.1186/s12864-016-2584-7 (PMC4804494; doi:10.1186/s12864-016-2584-7)
Supplement: Additional file 1: — Supplemental methods are more detailed descriptions of some of the computational methods used in EPIG-Seq. (DOCX 97 kb) [file 12864_2016_2584_MOESM1_ESM.docx]

EPIG-Seq: Extracting Patterns and Identifying Co-expressed Genes from RNA-Seq Data

Jianying Li^1,2,4^ and Pierre R. Bushel^2,3,*^

^1^Integrative Bioinformatics Group, ^2^Microarray and Genome Informatics Group, ^3^Biostatistics and Computational Biology Branch, National Institute of Environmental Health Sciences, Research Triangle Park, NC 27709

^4^Kelly Government Solutions, Research Triangle Park, NC 27709

**Supplemental Methods**

**Simulated RNA-Seq data**

Let $Y_{gs_{m}}$denote the count of a gene *g* $\in${1,…,*G*}, belonging to a group *m* $\in${1,…,*M*} of a sample *S*$\in${S_1_, S_2_,…,S_N_} such that:

$Y_{gs_{m}}$~NB( mean = $\mu_{gs_{m}}$, var=$\mu_{gs_{m}}$ ( 1 + $\frac{\mu_{gs_{m}}}{\theta_{gs_{m}}}$))

where, $\theta_{gs_{m}}$is the parameter measuring the dispersion in the data and $\mu_{gs_{m}}$is the true mean of the data. We set the dispersion parameter for each gene to be the same for all samples. Thus, $\theta_{gs_{m}}$= $\theta_{g}$. Here,

$$\mu_{gs_{m}}=E[Y_{gs_{m}}]=\frac{\lambda_{gs_{m}}}{\sum_{g=1}^{G} \lambda_{gs_{m}}}M_{s_{m}}$$

where, $M_{s_{m}}$is the sequencing depth for the *m*th group in sample *S*. $M_{s_{m}}$ = 10^7^$U_{s_{m}}$for $U_{s_{m}}$~Unif[0.7,1.4]. The estimation of the sample mean $\lambda_{gs_{m}}$and $\theta_{g}$ were obtained from a DESeq analysis of RNA-Seq level 2 count data from 10 randomly chosen human normal breast tissue samples obtained from The Cancer Genome Atlas (TCGA) downloaded from the National Cancer Institute (NCI) portal: <https://tcga-data.nci.nih.gov/tcga/dataAccessMatrix.htm>. The RNA-Seq data was produced on the Illumina GAII sequencer and analyzed by the TCGA team with SeqWare <https://seqware.github.io/> (Version=0.7.0) using RNASeqAlignmentBWAWorfklow (Version=0.7.10) and RNASeqQuantificationWorfklow (Version=0.7.2). Then, to generate simulated genes across groups of samples, we defined$\lambda_{gs_{m}}=\gamma_{gs_{m}}^{v_{gs_{m}}}\lambda_{gs_{1}}$, where S_1_ is the control (baseline) group of samples, S_m_ is a group of samples with a particular phenotype and .

The parameter $w_{gs_{m}}$ denotes the lower bound threshold of differential expression for the *g*th gene between S_m_ and S_1_. The differential expression was set at different levels for the respective groups using $v_{gs_{m}}$as an exponential value. The values of $v_{gs_{m}}$are denoted below.

|  |  | m |  |  |
| --- | --- | --- | --- | --- |
| g | 1 | 2 | 3 | 4 |
| Group A | 0 | 1 | 2 | 3 |
| Group B | 3 | 2 | 1 | 0 |
| Group C | 0 | 1 | 3 | 2 |
| Group D | 0 | 3 | 2 | 1 |
| Group E | 0 | 3 | 3 | 3 |
| Group F | 0 | 0 | 0 | 0 |

**Alignment of the TGxSEQC MOA data**

For each sample, a two-step alignment was performed using the fastq files. In the first step, raw reads were aligned with Novoalign v2.08.01 (www.novocraft.com) against rat genome rn4 downloaded from the University of California-Santa Cruz ftp server (ftp://hgdownload.cse.ucsc.edu/goldenPath/rn4). The intermediate BAM files generated with Novoalign were then parsed through customized scripts to summarize mapping results. Unmapped reads were passed to Novoalign again and were mapped to the rat RefSeq transcriptome gene model (release version 52, March 5, 2012) downloaded from the National Center for Biotechnology Information ftp server (ftp://ftp.ncbi.nih.gov/refseq). The alignment results were parsed through customized scripts to summarize mapping results and then merged with the results generated in step one.

**Maximization of *CY_d_***

*CY_d_* is maximized by assigning *x_ij_* = 1 and *x_kj_* = *D_k_* for *a*/2 times and likewise *x_kj_* = 1 and *x_ij_* = *D_i_* *a*/2 times. Here

$D_{i}=\frac{\sum_{j=1}^{a} x_{ij}-a/2}{a/2}$, $D_{k}=\frac{\sum_{j=1}^{a} x_{kj}-a/2}{a/2}$ for *x_ij_* ≥ 1 and *x_kj_* ≥ 1. Thus, maximum *CY_d_* = *D_1_* + *D_2_* + *D_3_*, where

$D_{1}=\sum_{j=1}^{b} \left(\frac{(x_{ij}+0.1)\log10\left(\frac{x_{ij}+0.1}{2}\right)-x_{ij}\log10(0.1)-(0.1)\log10x_{ij}}{x_{ij}+0.1}\right)$,

$D_{2}=\sum_{j=1}^{c} \left(\frac{(x_{kj}+0.1)\log10\left(\frac{x_{kj}+0.1}{2}\right)-x_{kj}\log10(0.1)-(0.1)\log10x_{kj}}{0.1+x_{kj}}\right)$,

$D_{3}=\left[\frac{a}{2}\left(\frac{(D_{i}+1)\log10\left(\frac{D_{i}+1}{2}\right)-\log10(D_{i})}{D_{i}+1}\right)+\frac{a}{2}\left(\frac{(D_{k}+1)\log10\left(\frac{D_{k}+1}{2}\right)-\log10(D_{k})}{D_{k}+1}\right)\right]$,

*b* is the total number of samples with read counts present in profile *i* only and *c* is the total number of samples with read counts present in profile *k* only.

**Magnitude of change**

The *g*th gene expression profile’s signal is:

$$S_{g}=\left\{\begin{matrix} \max\left\{z_{g}\right\},if\min\left\{z_{g}\right\} & > 0 & \\ -\min\left\{z_{g}\right\},elseif\max\left\{z_{g}\right\} & < 0 & \\ \max\left\{z_{g}\right\}-\min\left\{z_{g}\right\} & otherwise & . \end{matrix}\right.$$

When the sample size for each group is small, the approximated Z-statistic from the Wilcoxon rank sum test can be spurious. In such a case, we default to measure the strength of the *g*th gene’s differential expression according to the value of the Hodges-Lehmann location parameter estimator $\hat{\Delta}_{g}$ for the difference between two groups of independent samples [[23](#_ENREF_23)]. Briefly, $\hat{\Delta}_{g}$ is the median of all possible pairs of differences between the *g*th gene RNA-Seq counts in sample X (sample size = *n*) vs the RNA-Seq counts in sample Y (sample size = *m*). Thus, there will be nm differences. Hence,

$\hat{\Delta}_{g}=median\left\{x_{i}^{g}-y_{j}^{g},i=1,...,n, j=1,...,m\right\}$.

**Dispersion**

Count data is known to be dispersed. The variance-to-mean ratio (*VMR*) is a measure of dispersion ($\theta$) and is the inverse of the SNR. For Poisson distributed data, the variance is equal to the mean, i.e., V(*Y_g_*) = E(*Y_g_*) = *μ_g_*. If $\theta$is larger than 1, a data set is said to be overdispersed. The quasi-Poisson likelihood model is commonly used for overdispersed count data as it incorporates $\theta$ into the Poisson model such that that V(*Y_g_*) = *μ_g_θ_g_* .

**Count Data Modelling**

The variance of the response (*Y_g_*) is a linear function of the mean, V(*Y_g_*) = $\theta_{g}\mu_{g}$ and dispersion estimated as

where *n* is the sample size, *c* is the number of estimated parameters and$\hat{\mu}_{g}$is an inverse function of the linear predictors. Here, the inverse function is a “log” link in the form of a generalized linear model:

$Y_{gj}=\mu_{g}+X_{gj}+\varepsilon_{gj}$ , where for the *g*th gene expression profile in the *j*th sample, *Y* is the read count, *X* is the independent variable and *ε* is the random error term.

**Clustering of gene expression profiles to patterns**

During clustering, the $CY_{s_{ik}}$measure is used to correlate the *i*th gene profile to the *k*th pattern. The profile is assigned to the pattern to which it has the highest similarity to. Once all the profiles are assigned, a representative profile for each pattern is determined by choosing the highest median correlation to the other profiles in the pattern. Briefly, for the *i*th gene expression profile and for the *k*th pattern it is assigned to, a Pattern Correlation Score

$$PCS(x_{i})=\mathop{\text{median}}\limits_{j\in P_{k},j\neq i} \left\{corr(x_{i},x_{j})\right\}$$

is computed as the median of the correlations among the *i*th profile (*x_i_*) to all other profiles (*x_j_*) assigned to pattern *k* (*P_k_*). Until no more profiles are reassigned, the $CY_{s_{ik}}$ measure is used to correlate the *i*th profile to the *k*th pattern and assign it to the pattern with which it has the highest correlation. Since the *CY_s_* does not denote the directionality of the correlation, we restrict the assignment of a profile if and only if, corr(ind(Z_i_),ind(Z_j_)) for all groups = 1. Here corr() is the Pearson correlation and ind() is the indication of + or – of the location parameter Z.

The EPIG-Seq algorithm pseudo code

**Step 1: Extract candidate profiles as seeds to patterns**

1. Define the parameter set Ω for pattern extracting;
2. **for** i = 1..n **do**

Calculate all pairwise CY_s_ correlations

**end**

1. Delete the *i*th profile if the number of profiles with CY_s_ >= Rt1 is < Mt(set = 6)
2. For remaining profiles, estimate
   1. the location parameter
   2. the dispersion
3. Delete profile if location parameter < St1 or [dispersion < > 5% in each tail of the distribution]
4. Remove profile with max(dispersion) if the top 5 correlated profiles overlap with another profile
5. Remove correlated profiles that are redundant

Remaining *k* profiles are defined as candidate seeds for patterns

**Step 2: Cluster profiles to patterns**

1. Define the parameter set Ω for clustering;
2. **for** i = 1..n **do**

Initially assign the ith profile to the kth pattern if CY_s_ >= Rt2 and location parameter > St2

**end**

1. Until no more moves (when 1-AMI < 0.0001 or # of moves = 100)

**for** i = 1..m **do**

Update patterns with profiles with the highest median PCS

Assign the ith profile to the kth pattern if CY_s_ >= Rt2

**end**

1. Report final assignment of profiles to patterns

**Adjusted Mutual Information**

Let: $\text{MI}(A;B)=\sum_{a\in A} \sum_{b\in B} p(a,b)\log\left(\frac{p(a,b)}{Ent(a)Ent(b)}\right)$

be the mutual information between the two clustering results where, *p*(*a,b*) is the joint probability distribution of *A* and *B*, and Ent(*X*) is the entropy of clustering outcome *X*. Given *k* clusters,

$$Ent(X)=-\sum_{i=1}^{k} p_{i}\log_{2}(p_{i})$$

where *p_i_* is the probability of the *i*th cluster membership in clustering *X*.  To adjust the MI for chance occurrence,

$$AMI=\frac{MI-EMI}{(\max(Ent(a),Ent(b))-EMI)}$$

where, EMI is the expectation of the mutual information.  AMI ranges between 0 and 1.  An AMI value of zero means that the two clustering outcomes are independent whereas an AMI of 1 indicates that the two clustering are highly associated.

**Validation of Clusters**

Let *n_ij_* be the number of genes that are in both group *u_i_* and pattern *v_j_* of the *U* and *V* cluster partitions. Let *n_i_*. and *n_.j_* be the number of genes from group *u_i_* and pattern *v_j_* respectively and *n..* = *n*, the total number of genes. Assuming a hypergeometric distribution as the model of the *U* and *V* partitions being picked at random such that the number of genes in the groups and patterns are fixed, the Adjusted Rand Index is:

and ranges between 0 and 1. When two clustering partitions agree totally, *R*’ is 1 and when the partitions are selected by chance, *R*’ is 0.

**General silhouette for cluster validity**

The validity (optimization) of a clustering result is assessed using the general (average) silhouette [[25](#_ENREF_25)]. The higher the general silhouette value, the more cohesive (compact) the clusters are and the more separated individual clusters are from one another. The general silhouette of a clustering result is

$$\frac{1}{m}\sum_{i=1}^{m} \text{cluster\_silhouette}_{\text{i}}$$

where

cluster_silhouette = $\frac{\sum_{i=1}^{\left|\text{A}\right|} s(X_{i})}{\left|\text{A}\right|}$, $s(X_{i})=\frac{b(X_{i})-a(X_{i})}{\max\left\{a(X_{i}),b(X_{i})\right\}}$,

$a(X_{i})=\frac{\sum_{j\in\text{A},j\neq i} d(X_{i},X_{j})}{\left|\text{A}\right|-1}$, $d(X_{i},C)=\frac{1}{\left|C\right|}\sum_{j\in C} d(X_{i},X_{j})$, $b(X_{i})=\min_{\text{c}\neq\text{A}} \left\{d(X_{i},C)\right\}$,

*m* is the number of clusters, *C* and *A* are clusters, and the range of *s*(*X_i_*) is between –1 and +1.

**Searching the parameter space**

To extract patterns (step 1) and cluster gene profiles to the candidate patterns (step 2), user-defined parameters are set. To search for optimal parameter settings, in the first step, we fixed the parameters for profile clustering in step 2 with the *CY_s_* similarity at 0.8 and location parameter St2 at 2. Then, we searched the parameter space for step 1. These included: the *CY_s_* similarity [0.5 – 0.9] at interval 0.1, the location parameter St1 at [1 – 5] at interval 0.5 and the dispersion tail (on both sides) [1 – 5%] at interval 1%. With the parameter set for step 1 fixed, we searched the parameter space for step 2. These included: the *CY_s_* similarity [0.5 – 0.9] at interval 0.1 and St2 at [1 – 5] at interval 0.5. To evaluate the impact of the parameters on EPIG-Seq performance, we used the Adjusted Rand Index (*R’*) [[27-30](#_ENREF_27)] together with the true number of real patterns from the simulated data. *R’* ranges between 0 and 1. When two clustering partitions agree totally, *R*’ is 1 and when the partitions are selected by chance, *R*’ is 0. Further details of *R’* are available in the Supplemental methods.

**Comparing two clustering outcomes**

To assess the reproducibility of pattern extraction and subsequent clustering of the genes, we adopted a method for accessing clustering reproducibility [[31](#_ENREF_31)]. To compare two clustering results during the recursion and reallocation portions of the gene clustering to patterns part of EPIG-Seq (step 2), the mutual information (MI) between the two clustering results was used.  MI, when log base 2 is used, is interpreted in units of bits and is the reduction in uncertainty about the samples groupings in one clustering outcome given the other clustering outcome (i.e., clustering A vs clustering B). To adjust the MI for chance occurrence, an adjusted mutual information (AMI) measure is calculated based on the expectation of the mutual information. AMI ranges between 0 and 1.  An AMI value of 0 means that the two clustering outcomes are independent whereas an AMI of 1 indicates that the two clustering are highly associated. Further details of comparing two clustering outcomes are available in the Supplemental methods.

**Measuring the performance of clustering**

The performance of EPIG and EPIG-Seq is ascertained by measuring the sensitivity (the true positive rate) and specificity (the true negative rate) of the clustering results [[25](#_ENREF_25)].

Sensitivity = True positive/(True positive + False negative).

Specificity = True negative/(True negative + False positive).

For each known pattern, the denominator for the sensitivity computation is 200 (i.e, the number of simulated genes in a pattern), and the denominator for the specificity computation is 1000 (i.e, the total number of simulated genes in all five responsive patterns).
