# Supplementary figures and images for: EPIG-Seq: extracting patterns and identifying co-expressed genes from RNA-Seq data
Source: BMC Genomics. 2016 Mar 22;17:255. doi: 10.1186/s12864-016-2584-7 (PMC4804494; doi:10.1186/s12864-016-2584-7)

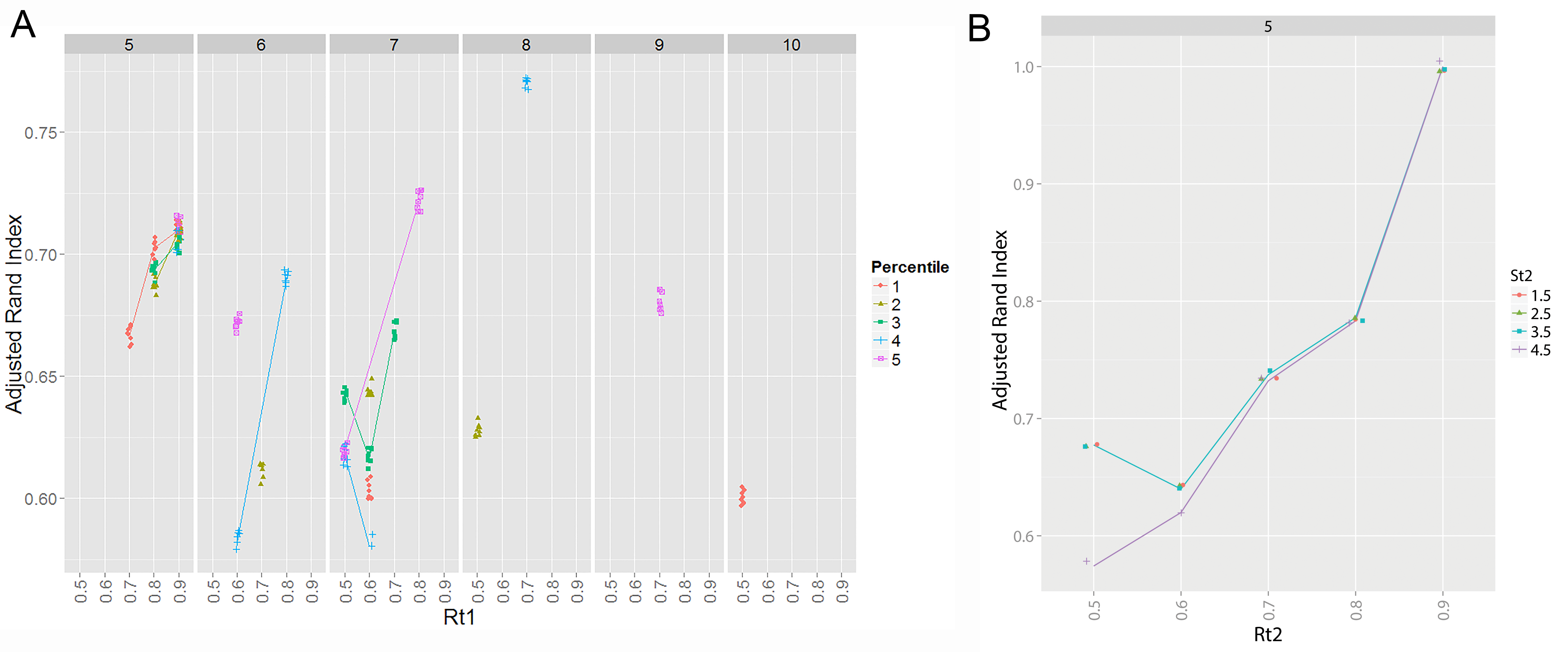

Supplement: Additional file 2: Figure S1. — A and B are tiff image files of the plots illustrating the optimization of parameters for EPIG-Seq steps 1 and 2 respectively using simulated data. (TIF 19517 kb) [file 12864_2016_2584_MOESM2_ESM.tif]

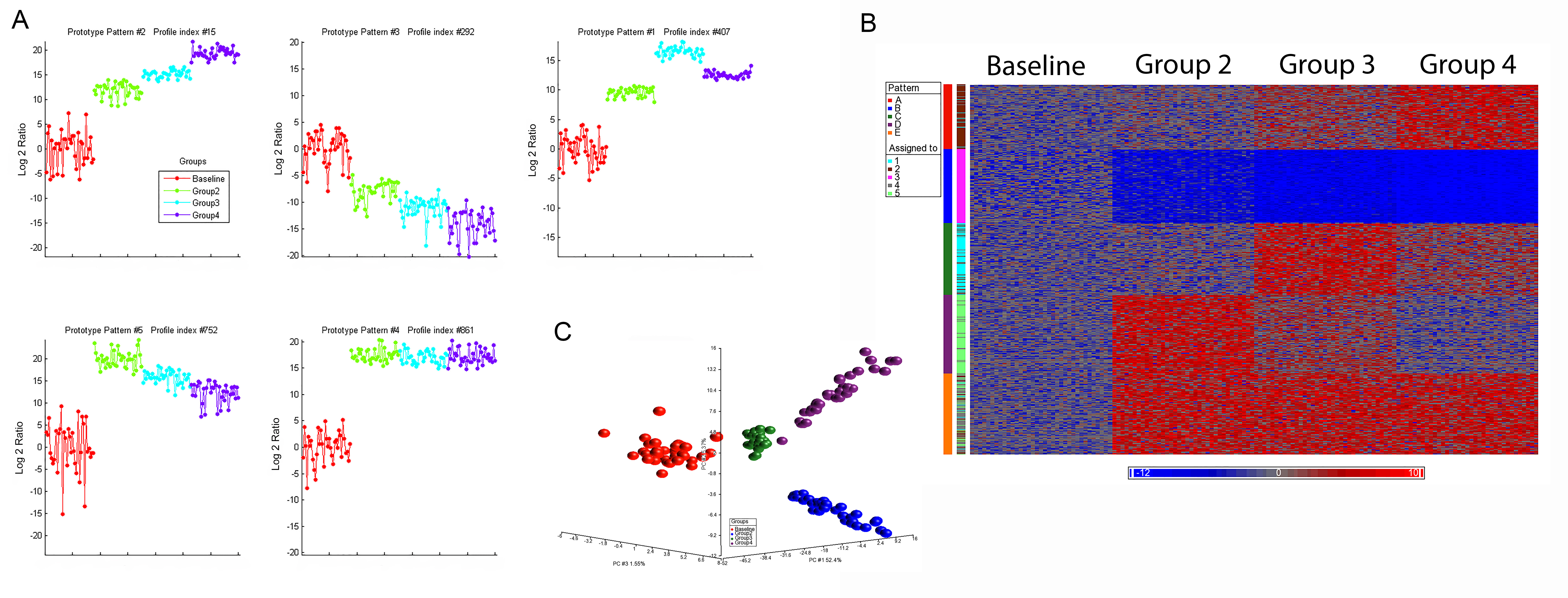

Supplement: Additional file 3: Figure S2. — EPIG-Seq analysis of the simulated data. A) Thumbnail plots of the simulated gene profiles that are the representatives (those with the highest pattern correlation score) of each of the extracted patterns. B) Heat map representation of the patterns extracted and the simulated gene profiles clustered to each. C) PCA of the simulated gene profiles clustered to the patterns by EPIG-Seq using the CYs correlation measures. (JPG 3477 kb) [file 12864_2016_2584_MOESM3_ESM.jpg]
